# Comparative transcriptome analysis reveals major genes, transcription factors and biosynthetic pathways associated with leaf senescence in rice under different nitrogen application

**DOI:** 10.1186/s12870-024-05129-x

**Published:** 2024-05-18

**Authors:** Yafang Zhang, Ning Wang, Chenggong He, Zhiping Gao, Guoxiang Chen

**Affiliations:** College of Life Sciences, Nanjing Normal University, Nanjing, 210023 China

**Keywords:** Transcriptome, Nitrogen application, Senescence, Phytohormones, Transcription factors

## Abstract

**Background:**

Rice (*Oryza sativa* L.) is one of the most important food crops in the world and the application of nitrogen fertilizer is an effective means of ensuring stable and high rice yields. However, excessive application of nitrogen fertilizer not only causes a decline in the quality of rice, but also leads to a series of environmental costs. Nitrogen reutilization is closely related to leaf senescence, and nitrogen deficiency will lead to early functional leaf senescence, whereas moderate nitrogen application will help to delay leaf senescence and promote the production of photosynthetic assimilation products in leaves to achieve yield increase. Therefore, it is important to explore the mechanism by which nitrogen affects rice senescence, to search for genes that are tolerant to low nitrogen, and to delay the premature senescence of rice functional leaves.

**Results:**

The present study was investigated the transcriptional changes in flag leaves between full heading and mature grain stages of rice (*O. sativa*) sp. *japonica* ‘NanGeng 5718’ under varying nitrogen (N) application: 0 kg/ha (no nitrogen; 0N), 240 kg/ha (moderate nitrogen; MN), and 300 kg/ha (high nitrogen; HN). Compared to MN condition, a total of 10427 and 8177 differentially expressed genes (DEGs) were detected in 0N and HN, respectively. We selected DEGs with opposite expression trends under 0N and HN conditions for GO and KEGG analyses to reveal the molecular mechanisms of nitrogen response involving DEGs. We confirmed that different N applications caused reprogramming of plant hormone signal transduction, glycolysis/gluconeogenesis, ascorbate and aldarate metabolism and photosynthesis pathways in regulating leaf senescence. Most DEGs of the jasmonic acid, ethylene, abscisic acid and salicylic acid metabolic pathways were up-regulated under 0N condition, whereas DEGs related to cytokinin and ascorbate metabolic pathways were induced in HN. Major transcription factors include ERF, WRKY, NAC and bZIP TF families have similar expression patterns which were induced under N starvation condition.

**Conclusion:**

Our results revealed that different nitrogen levels regulate rice leaf senescence mainly by affecting hormone levels and ascorbic acid biosynthesis. Jasmonic acid, ethylene, abscisic acid and salicylic acid promote early leaf senescence under low nitrogen condition, ethylene and ascorbate delay senescence under high nitrogen condition. In addition, ERF, WRKY, NAC and bZIP TF families promote early leaf senescence. The relevant genes can be used as candidate genes for the regulation of senescence. The results will provide gene reference for further genomic studies and new insights into the gene functions, pathways and transcription factors of N level regulates leaf senescence in rice, thereby improving NUE and reducing the adverse effects of over-application of N.

**Supplementary Information:**

The online version contains supplementary material available at 10.1186/s12870-024-05129-x.

## Background

Rice (*Oryza sativa* L.) is one of the most important food crops in the world, with more than half of the global population depending on rice as their main source of food, so rice production would need to meet the demands of population growth [1, 2]. Nitrogen is a large amount of nutrients necessary for plant growth and development, and is one of the basic elements of biomolecules such as amino acids, proteins, chlorophyll, plant hormones and nucleic acids [3, 4]. At present, over-application of nitrogen fertilizer is still the main problem facing rice production in China, which not only causes a decline in rice quality, but also leads to a series of environmental problems [5, 6].

Plants utilize nitrogen in the soil mainly in organic and inorganic nitrogen, as the main form of plant uptake of inorganic nitrogen and include nitrate nitrogen (NO_3_^−^) and ammonium nitrogen (NH_4_^+^). Plants in the long-term successional process gradually formed the high efficient utilization of nitrogen regulatory mechanisms [7, 8]. NO_3_^−^ taken up by roots from the soil is usually reduced to NH_4_^+^, and only then can it be utilized by the plant; the entire chemical reaction process takes place in roots, stems, and leaves, but is dominated by the leaves [9]. In addition, N can be stored transiently in plant above-ground tissues as amino acids, nitrates or proteins and subsequently retransported to provide nitrogen to growing rice seeds [5].

Numerous studies have shown that 90% to 95% of rice yield comes directly or indirectly from photosynthesis [10]. Leaf is the main organ of rice photosynthesis, and leaf area, photosynthetic rate, photosynthetic functional period, etc. are important indicators reflecting the photosynthetic production capacity of rice. leaf nitrogen content is closely related to photosynthetic intensity, which in turn affects the dry matter production capacity of the plant [11]. Nitrogen as an important limiting factor in rice yield formation, plays a role in rice yield formation by affecting the accumulation and distribution of dry matter and nutrients with different application rates [12]. Increased nitrogen application not only increases rice leaf area, but also facilitates the synthesis of macromolecules related to photosynthesis such as chlorophyll and Rubisco enzymes, and improves the photosynthetic rate [13]. However, too much nitrogen fertilizer will reduce the starch synthesis ability and the transfer of stored substances from leaves, stalks and other tissues to the seed grain capacity [14].

It was shown that different nitrogen dosages had significant effects on leaf morphology, photosynthetic rate and population quality, and that nitrogen application could promote the increase of leaf area, which in turn increased the area of light capture [15]. The flag leaves of rice have been identified as a major source of carbohydrate production due to their higher photosynthetic efficiency [8]. Studies have shown that more than 80% of the ^14^C in flag leaves is transported to grains [16]. Therefore, the balance of C and N in flag leaves and the allocation to the seed are considered to be the key factors for nitrogen utilization efficiency and yield in rice [17].

Leaf senescence is an effective strategy of adaptation to the environment that has developed over a long period of evolution [18]. In the nutrient growth stage, the leaf converts light energy into bioenergy and produces nutrients, and then carbon assimilation in the leaf is replaced by chlorophyll and macromolecular catabolism and gradually enters into the senescence stage, at which time nutrients continue to be transferred to the seed, and its role is changed from a "reservoir" to a "source" [19]. Timely leaf senescence is essential for source/store balance and crop yield stabilization [20]. Leaf senescence is closely related to nitrogen reutilization. Studies have shown that 90% of the nitrogen in seeds comes from the reutilization of nitrogen-containing complexes stored in the plant [21]. When nitrogen is deficient, a large amount of sugar accumulates in the cells during the early stages of leaf senescence in tobacco, barley, etc., and feedbacks inhibit physiological processes such as photosynthesis [22]. However, leaf senescence is not the result of carbon or nitrogen action alone, but the result of carbon and nitrogen balance regulation [23]. It has been reported that excessive application of nitrogen also accelerates leaf senescence, while moderate application of nitrogen helps to delay leaf senescence and promotes the production of photosynthetic assimilation products in leaves [24].

Effah et al. revealed that increased nitrogen fertilization caused reprogramming of photosynthesis, phytohormone signaling and several other biosynthetic pathways in wheat [25]. In a recent study of rice senescence, it was found that co-expression of NB-ARC protein RLS1 and RMC resulted in altered patterns of subcellular localization, triggering the cell death process and resulting in reduced activity of the antioxidant enzyme APX1 [26]. Zhang et al. found from the whole genome sequences of soybean roots, stems, leaves, flowers and seeds that GATA44 and GATA58 might be involved in the regulation of nitrogen metabolism in soybean under low nitrogen stress [27]. Jagadhesan et al. revealed the role of NLP transcription factor in low nitrogen stress resistance in rice [28]. However, there are fewer reports on the effects of nitrogen application in rice on genes, transcription factors and biosynthetic pathways involved in senescence. The discovery of genes for low-nitrogen tolerance in rice will contribute to the understanding of the molecular mechanisms of senescence and low-nitrogen tolerance in rice, as well as to the genetic improvement of rice. Therefore, to improve the potential of manipulating the rate and onset of senescence in rice, the present study profiled the transcriptional changes of flag leaves sampled from full heading stage to mature grain stage under conditions of no, moderate, and high N conditions to identify the genes, transcription factors, and signaling pathways involved under these conditions.

## Results

### Phenotype, N and photosynthetic pigments content of rice leaves under different N conditions

**Fig. 1:**
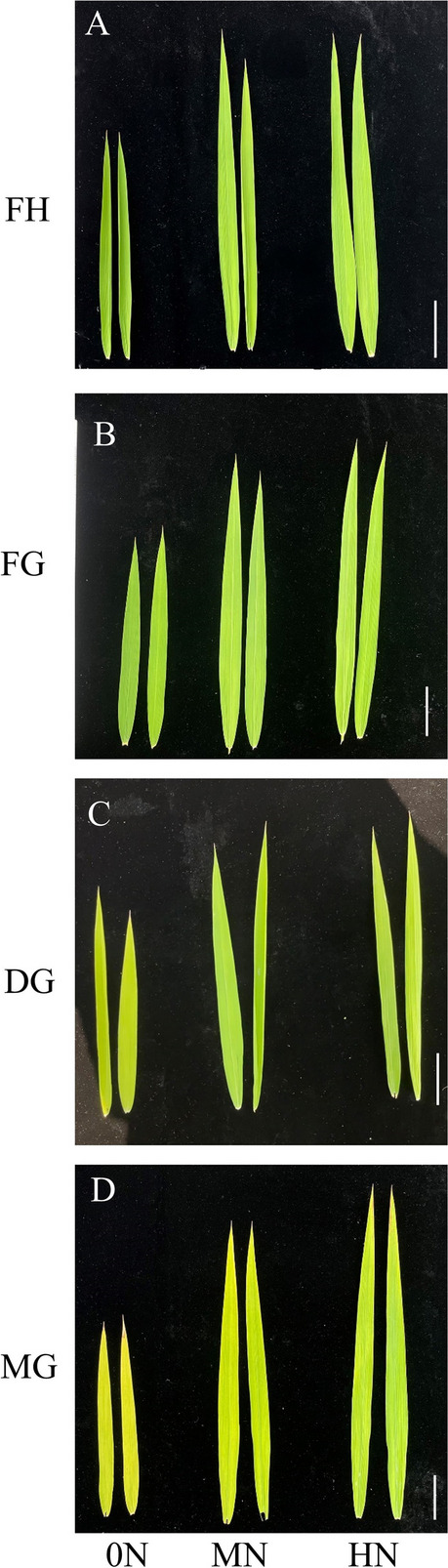
Photographs showing phenotypic changes of rice leaves under different levels of nitrogen fertilizer application between full heading stage and mature grain stage. **A** FH: Full heading stage; **B** FG: Filling grain stage; **C** DG: Dough grain stage; **D** MG: Mature grain stage. 0N: 0 kg/ha; MN: 240 kg/ha; HN: 300 kg/ha

As shown in Fig. 1, the growth of rice flag leaves was significantly weak under 0N condition, and the difference of growth status between MN and HN was not obvious. The difference of leaf color gradually appeared from dough grain stage, with leaves turning yellow under N-deficient condition and normal color under N-sufficient condition.

**Fig. 2:**
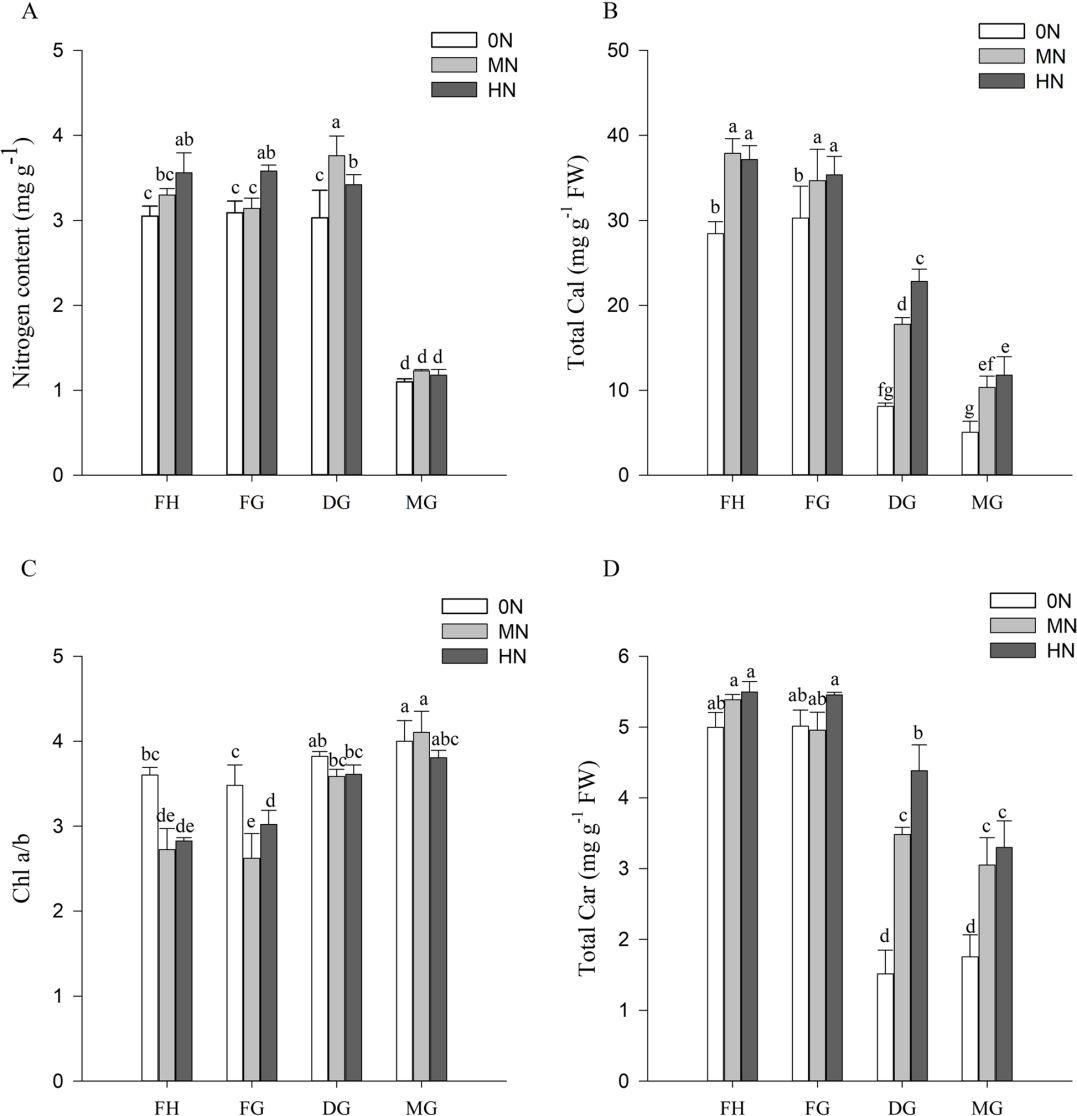
Nitrogen (N) and photosynthetic pigments contents of rice leaves under different levels of nitrogen fertilizer application between full heading stage and mature grain stage. **A** Nitrogen content; **B** Total Chl; **C** Chl a/b; D Total Car

In this study, we assessed the effect of three N application rates on leaf N content of rice cultivars. The results showed a positive correlation between N application and N content in leaves during full heading and filling grain stages, whereas during dough and mature grain stages, N content in leaves was higher in MN than HN condition (Fig. 2A), which indicated that excessive N application didn’t necessarily increase the rate of N translocation.

The photosynthetic pigment content of rice under different nitrogen conditions are shown in Fig. 2. The total Chl levels at full heading stage were 5.6, 3.7 and 3.2 higher than those at mature grain stage under 0N, MN HN, respectively (Fig. 2B). Total Car levels showed a similar trend. The total Car levels at full heading stage were 2.8, 1.8 and 1.7 higher than those at mature grain stage under 0N, MN HN, respectively (Fig. 2D). However, the Chl a/b ratio showed the opposite trend. The Chl a/b values gradually increased with rice growth and were significantly higher under N-deficient conditions than under N-sufficient conditions (Fig. 2C).

### Photosynthetic parameters of rice leaves under different N conditions

**Fig. 3:**
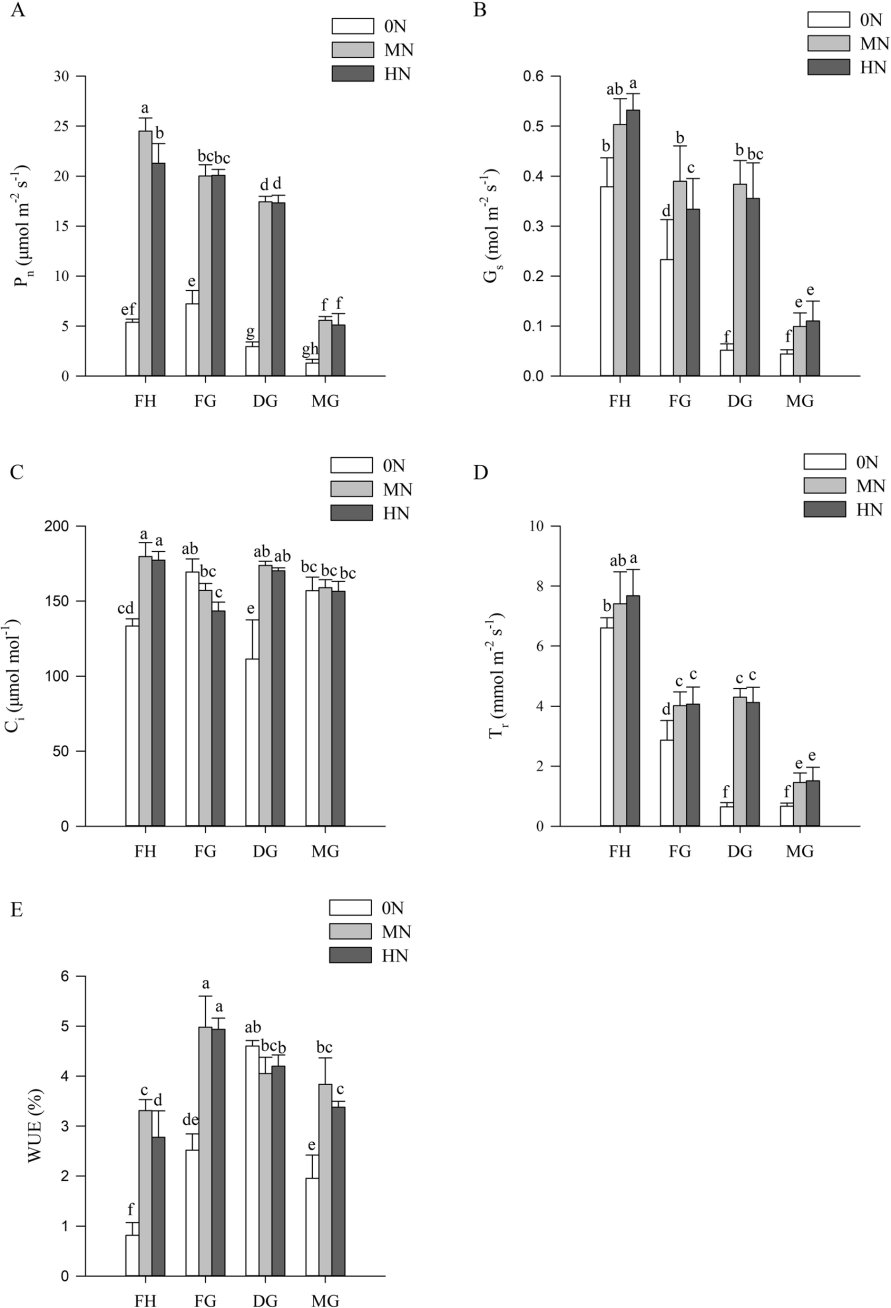
Photosynthetic parameters of rice leaves under different levels of nitrogen fertilizer application between full heading stage and mature grain stage. **A** P_n_: Net photosynthetic rate; **B** G_s_: Stomatal conductance; **C** C_i_: Intercellular CO_2_ concentration; **D** T_r_: Transpiration rate; E. WUE: Water use efficiency

The photosynthetic parameters of rice under different N conditions are shown in Fig. 3. The net photosynthetic rate (P_n_)under N-sufficient conditions was 2.8–5.9 times higher than that under N-deficient conditions, and this difference was most significant after the dough grain stage (Fig. 3A). Until mature grain stage, the stomatal conductance (G_s_) was reduced by 88.3%, 80.3%, and 79.3% under 0N, MN, and HN conditions, respectively (Fig. 3B), and the trend of transpiration rate (T_r_) was similar to it (Fig. 3D). The internal CO_2_ concentration (C_i_) was shown to be a non-stomatal limiting factor in the reduction of photosynthetic rate due to nitrogen deficiency at filling grain and mature grain stage (Fig. 3C). As rice matured, water use efficiency (WUE) declined more in N deficiency and more slowly in N sufficiency (Fig. 3E).

### Hormones content of rice leaves under different N conditions

**Fig. 4:**
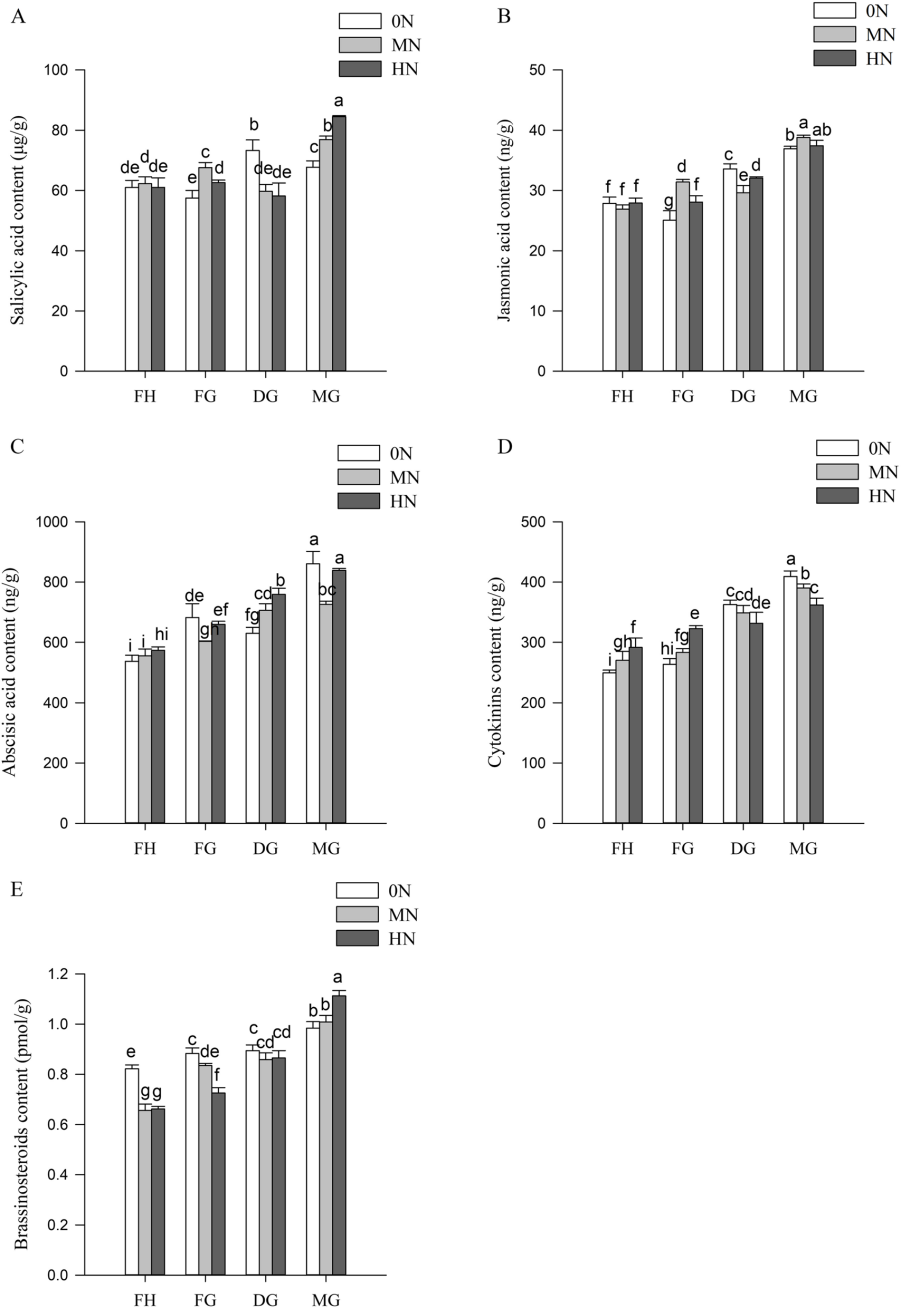
Hormones content of rice leaves under different levels of nitrogen fertilizer application between full heading stage and mature grain stage. **A** Salicylic acid; **B** Jasmonic acid; **C** Abscisic acid; **D** Cytokinins; E. Brassinosteroids

Salicylic acid content reached its highest value at dough grain stage under 0N conditions, whereas it peaked at mature grain stage under nitrogen-sufficient conditions (Fig. 4A). Jasmonic acid content varied significantly due to different nitrogen treatments after filling grain stage and gradually increased under 0N conditions (Fig. 4B). In general, abscisic acid content increased gradually and was lower under MN conditions among the different nitrogen treatments (Fig. 4C). Cytokinin content gradually increased with increasing nitrogen application while the trend was diametrically opposite from dough grain stage onwards (Fig. 4D). Brassinosteroids content was significantly elevated under nitrogen-deficient conditions and sharply elevated under nitrogen-sufficient conditions at mature grain stage (Fig. 4E).

### Transcriptome sequencing and assembly

To get more insights into the nitrogen-responsive mechanisms in rice, leaves samples of 0N, MN, and HN from four periods of rice in three biological replicates, were subjected to RNA sequencing via the Illumina sequencing platform. The summary of the transcriptomics data is presented in Table 1. The total clean reads generated varied from 43.81 to 76.28 million. The Q30 and GC content ranged 93.6–95.03 and 49.34–54.31%, respectively. A set of 604 transcripts length below 200 bp and sequence length of the longest transcript at 50% of the total transcripts number above 1,800 bp was obtained (Table S1). Transcripts are between 200-800 bp in length with about 6000 transcripts per 200 bp interval and the detailed length distribution of all transcripts is shown in Fig. S1. Among the total number of clean reads from the 36 samples in this study, 83.67% to 97.46% were successfully mapped against the assembled unigenes. Moreover, the unique mapping reads matching the sesame reference genome ranged from 81.09 to 94.54% (Table S2). We further subjected the FKPM of the three nitrogen concentrations samples at 4 stages to principal component analysis (PCA). Three biological replications were tightly grouped, and the three nitrogen concentrations of the four developmental stages separated from each other in the PCA plot (Fig. S2).

**Table 1:** Statistics of the sequencing for rice transcriptome

Sample	Clean reads	Error rate (%)	Q30 (%)	GC content (%)
FH_0N_1	50329498	0.0242	95	53.98
FH_0N_2	56941334	0.0249	94.28	54.15
FH_0N_3	52409938	0.0242	94.98	54.31
FH_MN_1	47686474	0.0248	94.38	54.17
FH_MN_2	51288656	0.0244	94.71	53.94
FH_MN_3	52586658	0.0246	94.5	53.96
FH_HN_1	55525066	0.0245	94.6	54.03
FH_HN_2	49787128	0.0244	94.75	53.35
FH_HN_3	53776066	0.0243	94.83	53.78
FG_0N_1	46699010	0.0243	94.9	52.34
FG_0N_2	47115674	0.0244	94.78	53.09
FG_0N_3	45128784	0.0243	94.9	52.12
FG_MN_1	58706588	0.0244	94.81	53.79
FG_MN_2	51291540	0.0247	94.44	53.53
FG_MN_3	55204304	0.0244	94.81	53.75
FG_HN_1	50067512	0.0244	94.72	52.62
FG_HN_2	48666832	0.0245	94.68	52.74
FG_HN_3	55965900	0.0247	94.43	52.02
DG_0N_1	52524718	0.0243	94.88	50.4
DG_0N_2	53952090	0.0242	94.95	50.18
DG_0N_3	49546018	0.0244	94.77	50.4
DG_MN_1	47424076	0.0248	94.38	50.9
DG_MN_2	48629710	0.0246	94.55	50.69
DG_MN_3	43807124	0.0245	94.66	50.97
DG_HN_1	51447500	0.0246	94.56	50.88
DG_HN_2	51538942	0.0248	94.35	51.31
DG_HN_3	60381832	0.0244	94.75	49.34
MG_0N_1	65007422	0.0244	94.74	52.62
MG_0N_2	66869118	0.0242	94.96	52.04
MG_0N_3	76278628	0.0241	95.03	52.12
MG_MN_1	56692606	0.0244	94.8	51.05
MG_MN_2	51143286	0.0247	94.51	50.6
MG_MN_3	62492172	0.0242	94.97	50.33
MG_HN_1	56991418	0.0247	94.48	52.54
MG_HN_2	53195166	0.0256	93.6	52.18
MG_HN_3	49951358	0.025	94.14	51.61

*FH* Full heading stage, *FG* Filling grain stage, *DG* Dough grain stage, *MG* Mature grain stage, *0N* Nitrogen application 0 kg/ha, *MN* Nitrogen application 240 kg/ha, *HN* Nitrogen application 300 kg/ha

### Functional annotation

**Fig. 5:**
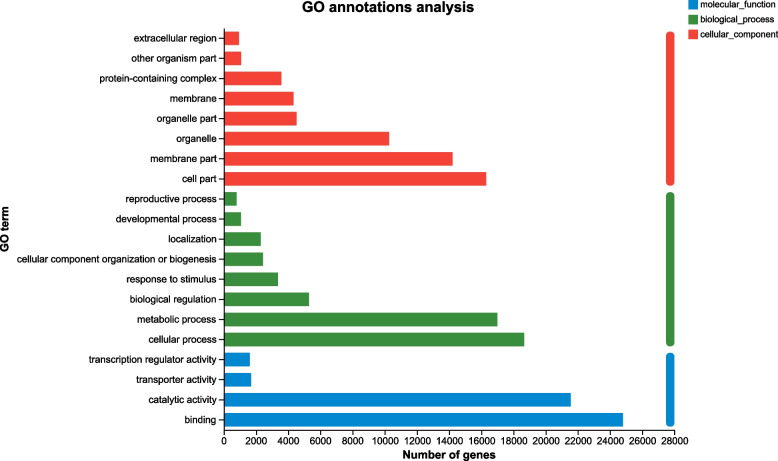
Gene Ontology (GO) classifcation of the assembled unigenes. Three main GO categories were shown in diferent colors (Green: Biological Process, Red: Cellular Component, Blue: Molecular Function). The GO terms name were shown at the vertical axis, the gene number were given at the horizontal axis

The unigenes were annotated using the NCBI GO, KEGG, COG, NR, Swiss-Prot, and Pfam databases. In this analysis, all unigenes exhibited high sequence similarity with known gene sequences (Table S3). Based on the GO annotation, 55986 unigenes were grouped into three functional GO categories, i.e., Cellular Component (CC; 26314), Biological Process (BP; 24345), and Molecular Function (MF; 34007), with subsets of sequences further divided into 8, 8, and 4 subcategories in these three groups, respectively (Fig. 5). There was a high representation of “cellular process” and “metabolic process” in the category BP, which included 18660 and 16995 unigenes in these subcategories, respectively. Furthermore, there was an enrichment of “binding” (24808 unigenes) and “catalytic activity” (21554 unigenes) in the MF parental category, and a high representation of “cell part” (16305 unigenes), “membrane part” (14209 unigenes), and “organelle” (10272 unigenes) in the CC category.

**Fig. 6:**
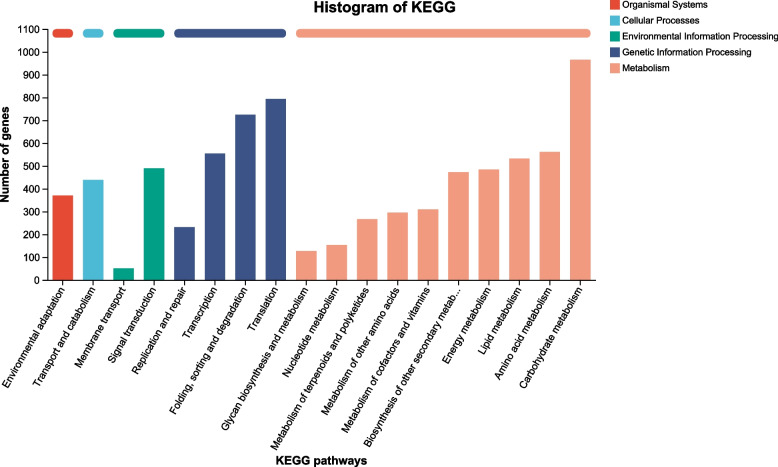
Histogram of KEGG pathway annotation of the unigenes in rice. The five main KEGG classifications were shown in different colors as shown at the right side. The x-axis and y-axis represent the annotated pathway and the number of annotated unigenes

To further understand the biological functions and interactions of the transcripts, a total of 12549 unigenes were mapped to 133 KEGG pathways and assigned to five major categories: Organismal Systems, Cellular Processes, Environmental Information Processing, Genetic Information Processing and Metabolism. The most abundant category was “carbohydrate metabolism”, followed by “translation”, “folding, sorting and degradation”, “amino acid metabolism”, and “transcription” (Fig. 6). These annotations and predicted pathways will aid the understanding of the nitrogen-responsive gene function in rice.

### Dynamic transcriptome changes in rice under different nitrogen levels

**Fig. 7:**
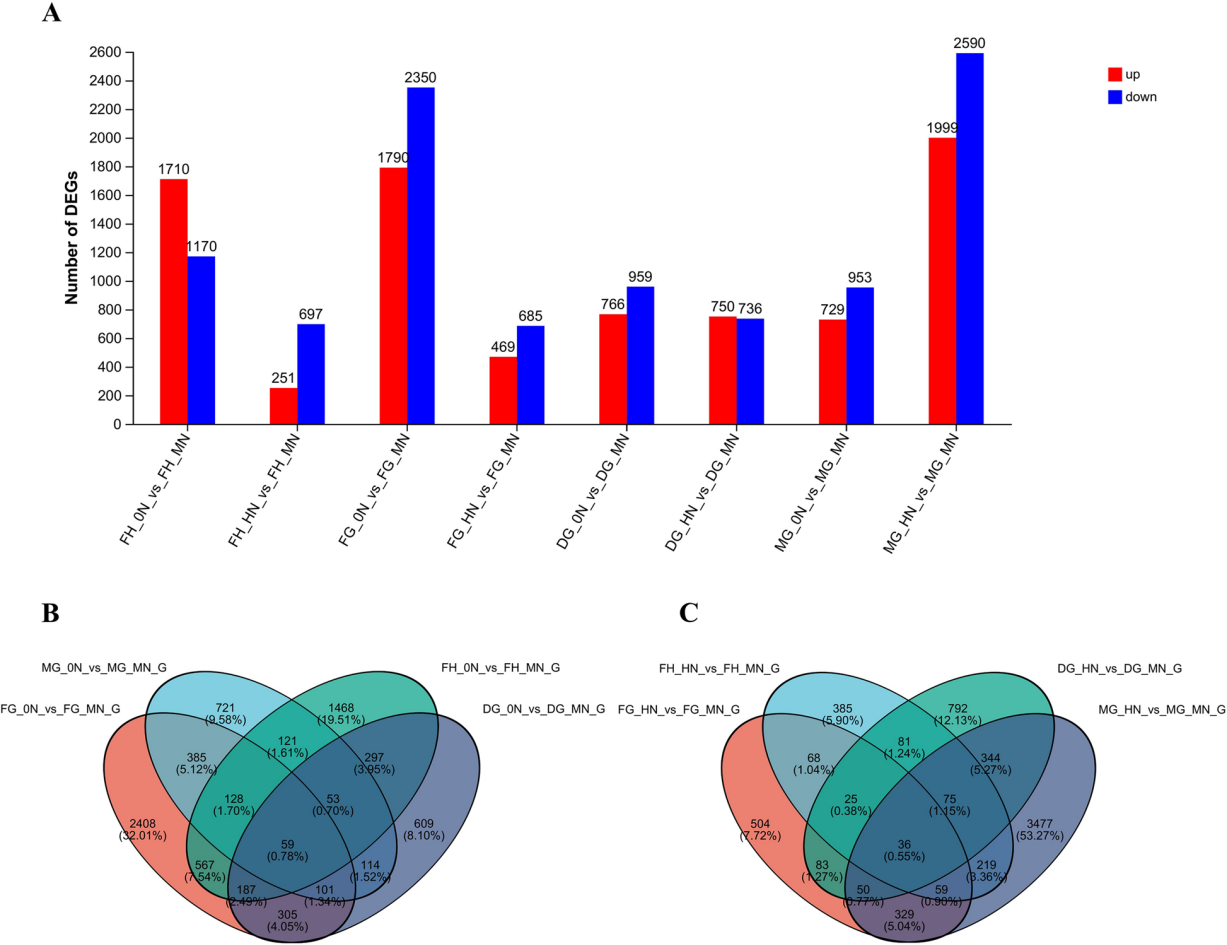
Differentially expressed genes (DEGs) along with different nitrogen application conditions between full heading stage and mature grain stage in rice. **A** Number of up- and down-regulated genes at different time points in rice. **B** Venn diagrams between DEGs at different time points for the 0N and MN comparison groups. **C** Venn diagrams between DEGs at different time points for the HN and MN comparison groups. respectively

To uncover nitrogen-induced changes in transcriptional levels in rice, we carried out differentially expressed genes (DEGs) analysis along with the nitrogen fertilizer application at various fertility stages. In total, we identified 10,427 DEGs, including 4,995 and 5,432 up- and down- regulated genes in no nitrogen, respectively. Meanwhile, 8,177 DEGs, including 3,469 and 4,708 up- and down- regulated genes, respectively, were identified in high nitrogen. In different nitrogen levels, the number of up-regulated DEGs showed opposite patterns with fertility, while the number of DEGs down-regulated under low and high nitrogen conditions was highest at filling grain and mature grain stage, respectively (Fig. 7A). We searched for genes that were significantly affected at all time points in different nitrogen levels. We detected 59 and 36 overlapped DEGs at the different time points in low and high nitrogen condition, respectively (Fig. 7B, C).

We selected DEGs with opposite expression trends under 0N and HN conditions for GO and KEGG analyses to reveal the molecular mechanisms of nitrogen response involving DEGs. The most GO terms that involve DEGs that up-regulated under N starved condition and down-regulated under N sufficient condition included hormone-mediated signaling pathway, response to temperature stimulus, protein folding, RNA modification and photosynthesis (Fig. S3A). The most GO terms that involve DEGs that up-regulated under N sufficient condition and down-regulated under N starved condition included protein folding, response to temperature stimulus, response to oxygen-containing compound, alpha-amino acid metabolic process and response to heat (Fig. S3B).

**Fig. 8:**
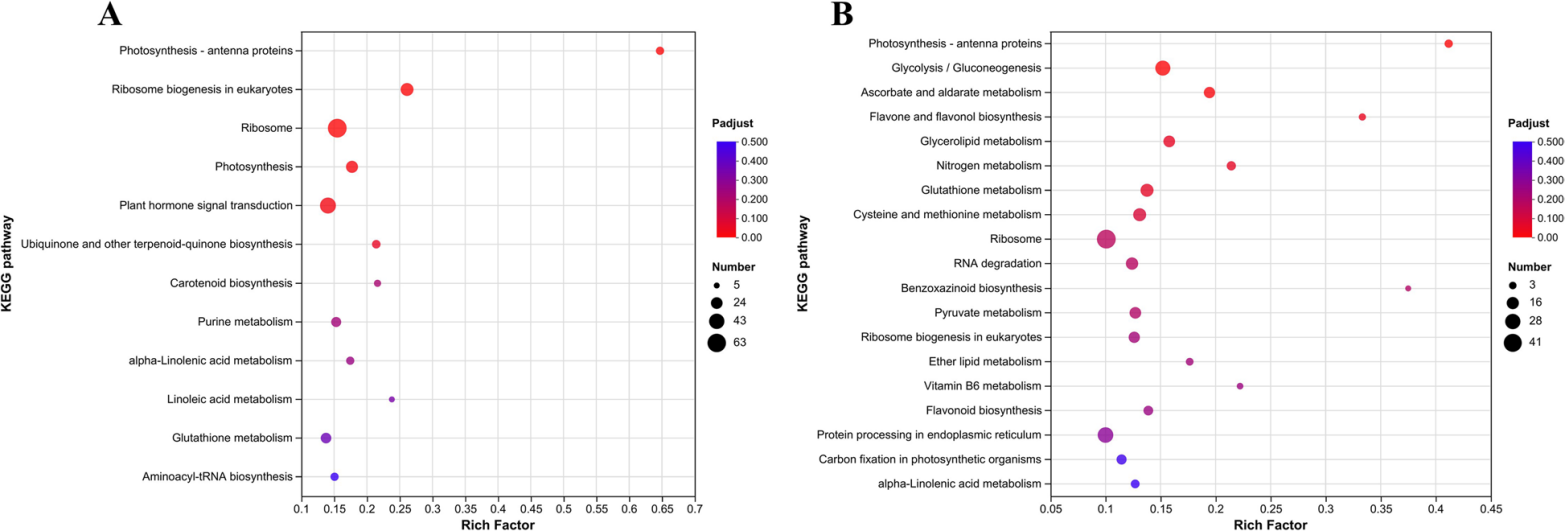
An overview of the KEGG pathways significantly enriched in DEGs. **A** KEGG pathway of DEGs that up-regulated in 0N and down-regulated in HN conditions. **B** KEGG pathway of DEGs that up-regulated in HN and down-regulated in 0N conditions

The KEGG analysis revealed that several pathways related to nitrogen response mechanisms were significantly induced during the fertility period. For instance, the DEGs that up-regulated under N starved condition and down-regulated under N sufficient condition were mainly assigned to ribosome, plant hormone signal transduction, ribosome biogenesis in eukaryotes and photosynthesis (Fig. 8A). the DEGs that up-regulated under N sufficient condition and down-regulated under N starved condition were mainly enriched to glycolysis / gluconeogenesis, ascorbate and aldarate metabolism and Photosynthesis-antenna proteins (Fig. 8B).

### DEGs participate in plant hormone signal transduction

**Fig. 9:**
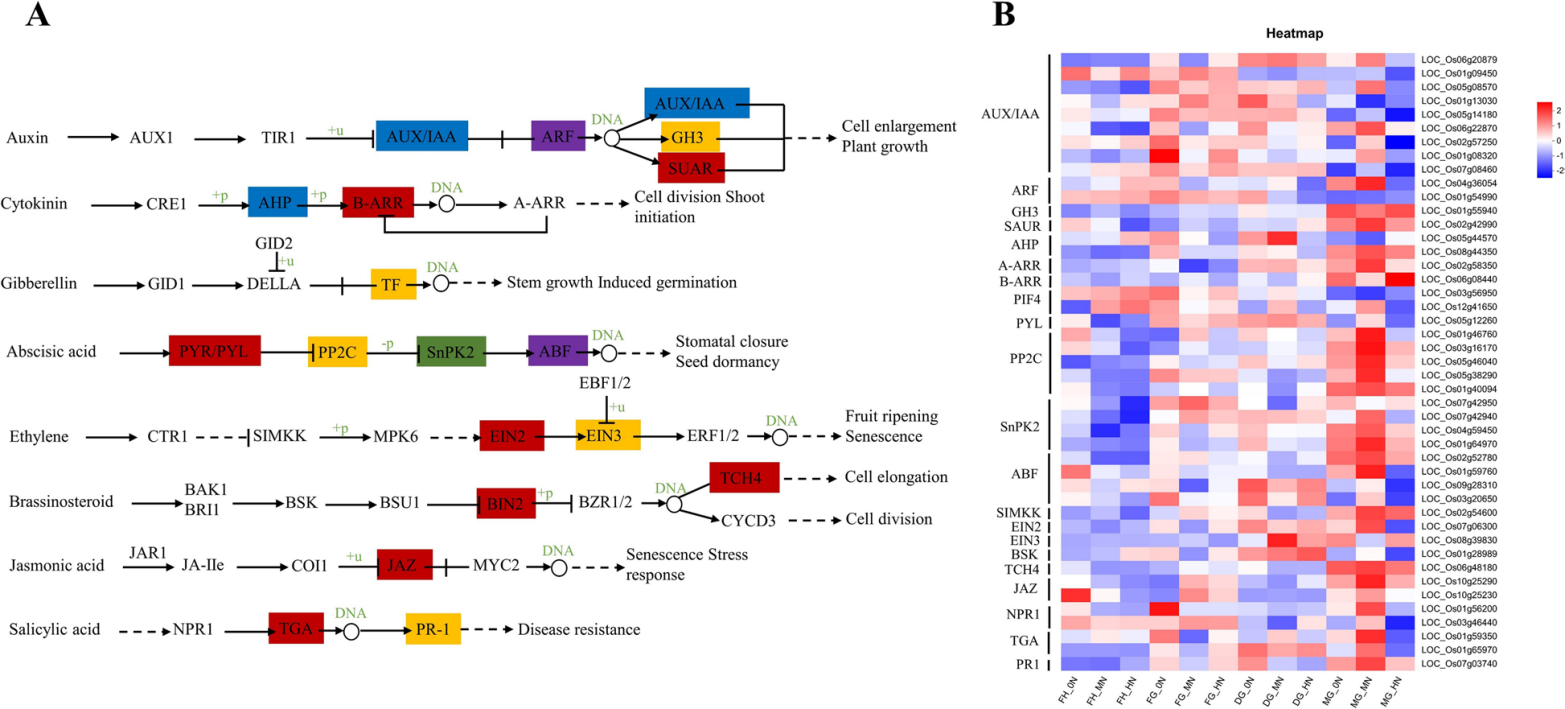
Significantly enriched plant hormone signal transduction in the KEGG pathway. **A** Schematic diagram of plant hormone signal transduction involved with differentially expressed genes in the current study. **B** Key enzymes expressed in our study have colored shaded rectangles and genes link to these enzymes as well as their expression are presented in the heatmap

Phytohormones play a crucial role in plant adaptation to adverse conditions, and the main hormones that regulate plant responses to abiotic stresses are gibberellin, abscisic acid, ethylene, jasmonic acid, and salicylic acid [29]. A total of 13 genes comprising 9, 2, 1 and 1 linked to auxin-responsive protein IAA (AUX/IAA), auxin response factor (ARF) as a transcription factor regulating the expression of growth hormone response genes, auxin responsive Gretchen Hagen 3 (GH3) gene family and Small Auxin Upregulated RNA (SAUR) family protein in the early response to auxin (Fig. 9). Compared to MN, Majority of these genes were upregulated in 0N and down-regulated in HN. For instance, LOC_Os01g08320 expressed 2.51 and 1.12 fold change in 0N and HN, respectively, but could not expressed in MN. In addition, LOC_Os04g36054, LOC_Os01g54990 (ARF) were mostly detected in 0N compared with HN. LOC_Os01g55940 (GH3) expressed higher in 0N and HN than MN. LOC_Os05g08570, LOC_Os01g13030, LOC_Os05g14180 and LOC_Os02g57250 expressed higher in 0N than either MN or HN. The results on auxin signal transduction suggest that N deficiency induced AUX/IAA, ARF, SAUR and GH3 genes.

Dissimilar to above, N concentration induced 4 genes in cytokinin signaling pathway with 2 histidine-containing phosphotransfer peotein (AHP) and 2 Arabidopsis response regulators (ARR) in the former whereas the latter B-ARR involved a gene LOC_Os06g08440 that is significantly up-regulated in HN. Phytochrome-interacting factor 4 comprised 2 genes exhibit similar expression patterns in gibberellin signaling (Fig. 9).

Abscisic acid signaling had the highest number of genes (14) link to abscisic acid receptor PYR/PYL family (PYL), PP2C-type protein phosphatase (PP2C), sucrose non-fermenting-1-related protein kinase 2 (SnRK2), ABA-responsive-element binding factor (ABF) with 1, 5, 4 and 4 genes respectively (Fig. 9) of these, were mostly expressed under 0N or MN conditions. The results indicate that N deficiency in rice induced gene expression in the abscisic acid signaling pathway.

In addition to the above, 4 structural genes involved in ethylene signal transduction mitogen-activated protein kinase kinase 4/5 (MKK4/5) and ethylene-insensitive protein 2 and 3 (EIN2 and EIN3) were significantly induced by 0N and MN during mature grain stage (Fig. 9). Similar trend was observed for LOC_Os06g48180 link to xyloglucosyl transferase TCH4 (TCH4) and the opposite in BR-signaling kinase (BSK) in brassinosteroid metabolism (Fig. 9).

Similarly, 2 structural genes mainly jasmonate ZIM domain-containing protein (JAZ) involve in jasmonic acid signal transduction mostly increased expression in N dearth (Fig. 9). 2 TGA genes, 2 regulatory protein (NPR1) and 1 pathogenesis-related protein 1 (PR-1) involve in salicylic acid metabolism expressed higher in low N condition relative to high N condition (Fig. 9). These suggest that the structural genes involved in jasmonic acid and salicylic acid signal transduction may be responsible for the increased low N content.

In summary, the above results indicate that rice reprogram a number of phytohormones in responding to either 0N or HN condition. Specifically, under 0N, ARF, IAA, ABF, EIN, JAZ, TGA, and NPR1 genes involve in auxin, abscisic acid, ethylene, jasmonic acid and salicylic acid signal transduction, respectively, were induced in order to survive, accumulate and remobilize N whereas under HN, cytokinin and brassinosteroid signal transduction may have accounted for the increased remobilized N compared to the N shortage conditions.

### DEGs participate in photosynthesis, glycolysis/gluconeogenesis, ascorbate and aldarate metabolism

A total of 37 genes were associated with photosynthesis as well as photosynthesis-antenna proteins (Table S4, Table S5 and Fig. S4A). All with the exception of LOC_Os06g39708 involve in photosystem II CP47 chlorophyll apoprotein (psbB) and LOC_Os01g57962 involve in photosystem I P700 chlorophyll a apoprotein A2 (psaB) expressed higher in the N deprivation condition compared to either of N abundant conditions. These suggest that rice repairs damage to chlorophyll due to nitrogen deficiency under low-nitrogen condition by inducing many photosynthesis structural genes.

Similarly, 26 DEGS were enriched in the glycolysis/glycolysis pathway associated with 15 enzymes of known function (Table S4, Table S5 and Fig. S4B). Most of these genes were up-regulated under both 0N and HN conditions and were significantly up-regulated under HN condition in mature grain stage, while two genes were highly expressed only under low nitrogen condition, among them include 1 pyruvate kinase (PK: LOC_Os01g47080) and 1 dihydrolipoyl dehydrogenase (DLD: LOC_Os03g45990).

In addition, there are 14 genes involved in ascorbate and aldarate metabolism that have inconsistent expression patterns (Table S4, Table S5 and Fig. S4C). LOC_Os01g62860, LOC_Os01g62870, LOC_Os01g62880 (AKR1A1), LOC_Os03g39000 (VTC4) and LOC_Os07g09330 (IMPL2) were mostly detected in either 0N or HN compared with 0N which only LOC_Os06g36560 (MIOX). 4 genes including LOC_Os05g02530 (DHAR), LOC_Os02g54890 (GAE), LOC_Os08g41090 and LOC_Os09g39380 were significantly expressed only in N shortage condition.

### Response of differentially expressed TFs to N rates

**Fig. 10:**
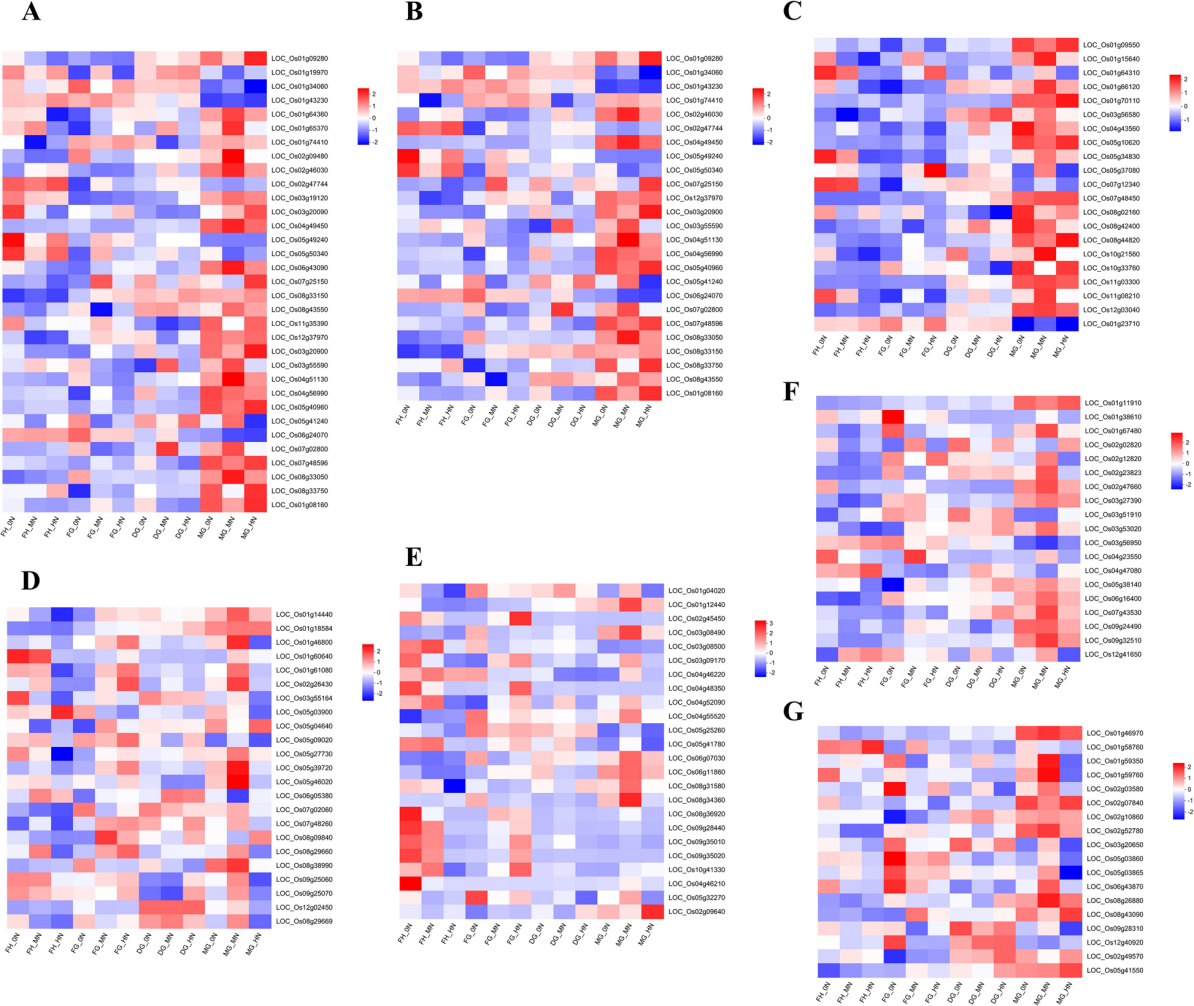
DEGs encoded for major transcription factor families. **A** MYB **B** MYB-related **C** NAC **D** WRKY **E** ERF **F** bHLH G. bZIP

Transcription factors (TFs) are a class of proteins that exercise their biological functions by regulating the transcription of target genes, and play a key role in plants to stressed condition [30]. We analyzed TFs derived from DEGs under different nitrogen conditions, which belong to 30 different expressed transcription factor families (Table S6). The major transcription factor families include MYB (33 genes), MYB-related (25 genes), ERF (24 genes), WRKY (23 genes), NAC (21 genes), bHLH (19 denes) and bZIP (18 genes) with varied levels of regulation of DEGs (Fig. 10).

Most of the genes in these three TF families (MYB, MYB-related and bHLH) have similar expression patterns under low and high nitrogen. This implies that these three TF families can be both positively and negatively regulated. In contrast, ERF, WRKY, NAC and bZIP TF families have similar expression patterns under 0N and MN suggesting that these TFs play crucial roles in transcriptional regulation in response to nitrogen.

### qRT-PCR validation

**Fig. 11:**
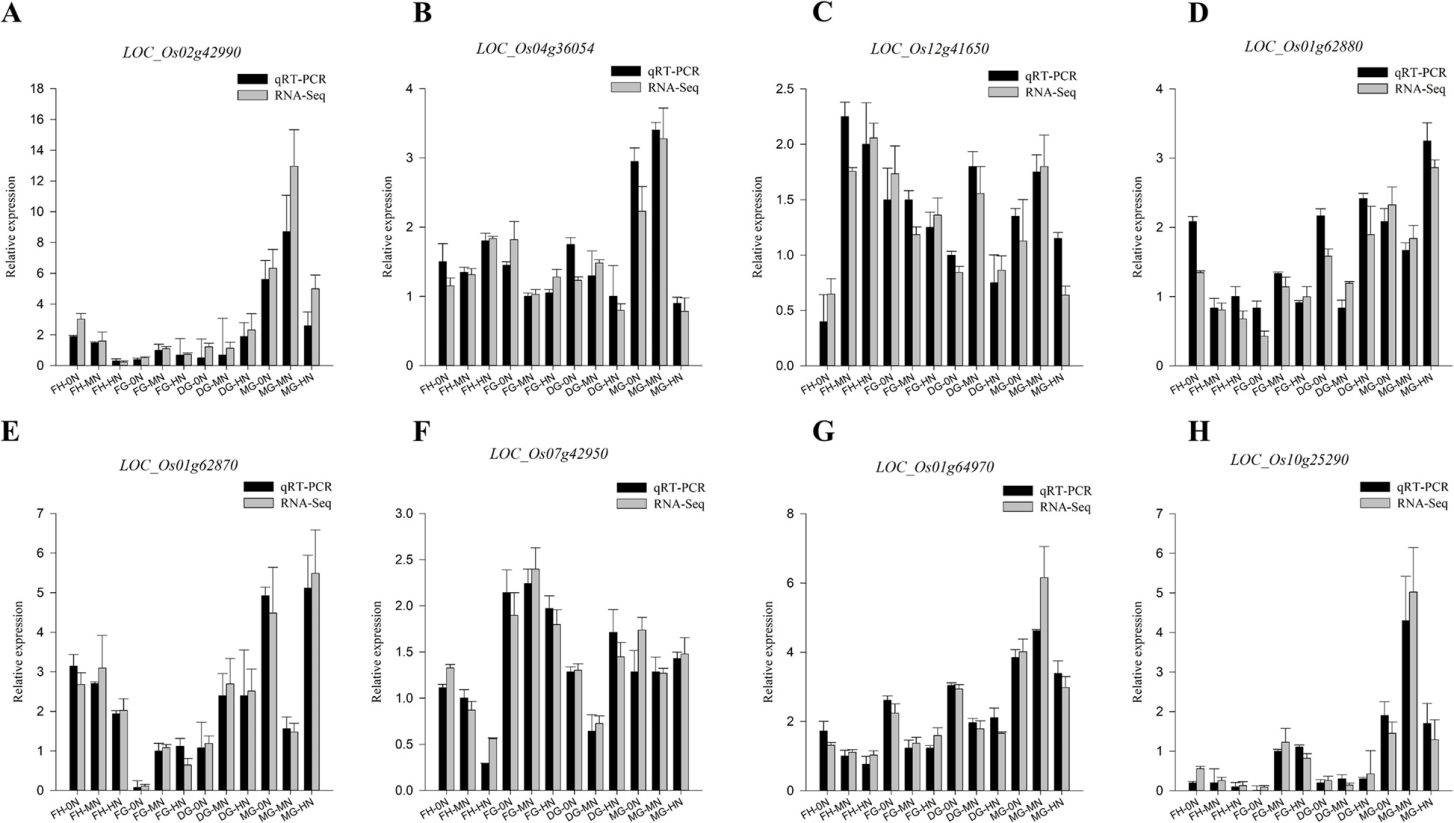
Validation of eight differentially expressed genes in rice by qRT-PCR

To validate the results of RNA-seq, 8 DEGs from some significantly enriched pathways were selected for qRT-PCR. The results of this experiment showed that the expression patterns of the RNA-Seq results were consistent with the qRT-PCR validation results (Fig. 11), indicating that the transcriptome data were accurate and reliable.

## Discussion

### N content of rice leaves under different N conditions

In recent decades, the application of large amounts of N fertilizer in agricultural production has become an important measure to increase crop yields, but crop nitrogen use efficiency (NUE) has not increased as a result [31]. Therefore, improving NUE is an effective strategy for sustainable agricultural development [32]. Studies have shown that about 75% of the N is enriched in chloroplasts during the early stages of growth, so the N content of leaves has an important effect on its photosynthesis [33]. Leaf senescence is the result of N reuse, which can provide the required N source for seed growth and development, and delayed senescence is a favourable measure to prolong the time of seed filling to obtain high yields [34]. The results of this study show that N content of leaves in dough and mature grain stage was highest in MN treatment (240 kg/ha) followed by HN treatment (300 kg/ha), respectively, and the least were observed in 0N (0 kg/ha) (Fig. 2A). These results suggest that there is not necessarily a direct causal relationship between high N application and N transport to leaves [35].

### Photosynthetic parameters and pigments of rice leaves under different N conditions

The phenotype of rice showed significant differences in flag leaves growth under different nitrogen treatments (Fig. 1). It has been shown that plants under low nitrogen conditions inhibit leaf growth by reducing the water potential for leaf expansion [36]. Leaf expansion is associated with P_n_, enabling rice to maintain photosynthesis under low nitrogen conditions [37]. In addition, nitrogen application optimized water use strategies in rice and improved WUE (Fig. 3). Shangguan et al. concluded that nitrogen levels were positively correlated with WUE and nitrogen application could promote above-ground growth [38]. In the present study, low nitrogen caused significant reduction in total Chl and Car content (Fig. 2). It indicated that N deficiency affected the absorption and capture of light energy in rice [39], Carotenoids counteracted photoinhibition through xanthophyll cycle, thereby reducing chlorophyll degradation [40]. The elevation of Chl a/b under N-deficient condition suggested that rice resisted low nitrogen stress by optimizing pigment ratios [41].

### Major biosynthetic pathways and genes involved in leaf senescence in rice under different N application

Plant leaf senescence is the result of a combination of internal gene expression regulation and external environment, and timely leaf senescence is important for ensuring rice yield [42]. In this study, DEGs responsive to N were found to be significantly enriched in plant hormone signal transduction (Fig. 8). It is well known that plants regulate the process of leaf senescence by inducing changes in hormone levels [18]. It is currently believed that hormones such as ethylene, ABA, SA, JA and BR promote plant leaf senescence, whereas cytokinin and gibberellin suppress leaf senescence [43], the results of the present study are not entirely consistent. Most of the genes involved in the abscisic acid signaling pathway using carotenoid as a precursor substance are up-regulated under 0N and MN conditions. ABA induced leaf senescence begins with disruption of chloroplast structure, chlorophyll degradation and reduced photosynthesis [44]. ABA-OsPYL-OsPP2C is a signaling pathway that has been shown to promote leaf senescence by ABA [45].

Ethylene is well known as a senescence hormone, and it regulates leaf senescence and abscission [46]. In this study, 3 ethylene insensitive genes (MKK4/5: LOC_Os02g54600; EIN2: LOC_Os07g06300; and EIN3: LOC_Os08g39830) were significantly expressed in 0N and MN conditions (Fig. 9). Recent studies have confirmed that EIN2 is one of the core components of the ethylene signaling pathway, and its loss-of-function mutant ein2 exhibits a delayed leaf senescence phenotype; EIN3, a transcription factor located downstream of EIN2, can directly bind to the promoter of the senescence-associated gene ORE1 and positively regulate its expression, and ethylene promotes Arabidopsis leaf senescence through the cascade of signaling mediated by EIN2-EIN3-ORE1 [47].

ARR is a key gene for cytokinin signaling, and A-ARR is a negative regulator of cytokinin signaling, which can inhibit the transcription of B-ARR by competing for arabidopsis his phosphotransfer protein (AHP), and exerts the function of negative feedback regulation of cytokinin signaling, B-ARR activates the expression of cytokinin-responsive genes and functions as a positive regulator of cytokinin signaling [48]. Consistent with the above, the present study found that B-ARR and A-ARR involved genes with opposite expression patterns under different nitrogen concentration conditions, B-ARR involved a gene LOC_Os06g08440 that is significantly up-regulated in 0N and HN (Fig. 9). It has been reported that cytokinin and nitrogen uptake and utilise together play an important role in leaf senescence, and that root cytokinin can be induced by nitrogen synthesis and transported to aboveground tissues to inhibit leaf senescence [49]. B-ARR delays senescence and improves tolerance in Arabidopsis thaliana [50]. Therefore, the present study speculates that cytokinin may exert molecular functions through B-ARR in regulating senescence delay and low nitrogen tolerance.

Ascorbic acid is involved in plant senescence regulation by regulating the expression of SAG [51]. In this study, we found that VTC4 involved gene LOC_Os03g39000 was significantly induced under HN condition in mature grain stage (Table S4 and Fig. S4). It has been demonstrated that the Arabidopsis *vtc-1* mutant induces an increase in the contents of ABA, SA, and ethylene, which in turn promotes the up-regulation of SAG expression and ultimately leads to senescence [52]. Arabidopsis *vtc4-1* mutant has half the ascorbic acid content of WT and has a premature senescence phenotype [53]. Therefore, rice in this study may have delayed senescence by inducing VTC4-related gene under HN condition. More studies have shown that MIOX regulates ascorbic acid synthesis and plays an important role in abiotic stresses such as drought and low nitrogen tolerance [54, 55]. In this study, MIOX was found to be significantly induced under MN and HN conditions. It is surmised that under N sufficient conditions, the MIOX-related gene is significantly induced to increase ascorbic acid synthesis and thereby retard senescence.

In addition to these, rice under 0N promotes the accumulation and catabolism of sugars and their related substances, regulates osmotic potential, and provides energy and precursors for the synthesis of secondary metabolites to the plant body, and also delayed senescence under HN condition through the regulation of glycolysis / gluconeogenesis pathway. DEGs involved in photosynthesis and glycolysis / gluconeogenesis pathways can also be used as target genes to delay senescence under N deficiency condition, thereby minimize N application in rice without affecting yield.

### Major TFs involved in leaf senescence in rice under different N application

TFs can activate or inhibit gene transcription and regulate gene activity and expression [56]. We detected 30 TF families regulating gene expression from DEGs responsive to N in the present study (Table S6). The major transcription factor families include MYB, MYB-related, ERF, WRKY, NAC, bHLH and bZIP with varied levels of regulation of DEGs (Fig. 10). These TFs have been shown to be involved in leaf senescence regulation and N remobilisation in previous studies [57–60]. We found that ERF, WRKY, NAC and bZIP TF families have similar expression patterns under 0N and MN, suggesting that these TFs can promote leaf senescence. *OsNAC5* expression is up-regulated during rice maturation, is regulated by ABA, and plays a role in senescence-associated nutrient reactivation by directly or indirectly controlling phloem sodium biosynthesis and metal translocation [61]. *OsWRKY42* induces senescence in rice leaves by repressing the expression of the reactive oxygen scavenger gene *OsMT1d* [62]. *BrERF72* is induced by methyl jasmonate and up-regulates jasmonic acid synthesis gene expression thereby accelerating leaf senescence via the jasmonic acid signaling pathway [63]. The results of this study provide a basis for leaf senescence in rice in order to optimise yield while minimising N application.

## Conclusion

This study has showed the usefulness of the transcriptomic approach in identifying DEGs in rice under varying N conditions by profiling the transcriptional changes induced by 0N, MN and HN in understanding the regulation of leaf senescence by nitrogen levels. We confirmed that different N applications caused reprogramming of plant hormone signal transduction, glycolysis/gluconeogenesis, ascorbate and aldarate metabolism and photosynthesis pathways in regulating leaf senescence. Most of the genes involved in the abscisic acid signaling pathway using carotenoid as a precursor substance are up-regulated under 0N and MN conditions. The genes detected in the ascorbate and aldarate metabolism pathway (VTC4 and MIOX) can delay leaf senescence in rice under N sufficient condition. We also found that ERF, WRKY, NAC and bZIP TF families can promote leaf senescence under 0N and MN conditions. The results provided new insights into the gene functions and pathways of N level regulates leaf senescence in rice, thereby improving NUE and reducing the adverse effects of over-application of N.

## Methods

### Plant materials and treatments

Rice (*O. sativa*) sp. *japonica* ‘NanGeng 5718’ was used in this study, which was cultivated at Jiangsu Academy of Agricultural Sciences. N fertilizer at four levels 0, 240 and 300 kg/ha designated 0N, MN and HN, respectively, were applied on each plot before planting. Base fertilizer, tiller fertilizer and spike fertilizer in the ratio of 4:3:3. Flag leaves were collected in liquid nitrogen after measurement of photosynthetic parameters in the field. The experiment was arranged in randomized complete block design with 3 replicates. Sword Leaves from five individual plants in 0N, MN and HN were sampled at full heading stage, filling grain stage, dough grain stage and mature grain stage and were divided in two samples; one sample was used to quantify N contents and the other one was immediately frozen in liquid nitrogen. Thirty-six samples, including the leaves sampled at full heading stage, filling grain stage, dough grain stage and mature grain stage from 0N, MN and HN were used for transcriptome analysis. Each sample was analyzed in triplicate.

### N content analysis

Take the same part of the leaf and put it in the oven at 110°C for 30 min, and then dry it at 70°C to constant weight. The nitrogen content of leaves was determined using the Elementar vario MACRO CUBE⁃CHNS mode [64]. The statistical analyses were performed using SigmaPlot 12.0 and IBM Statistics7.0 software.

### Phenotype and determination of photosynthetic pigments content

For each treatment, two flag leaves in the same state of growth were selected and photographed. The determination of photosynthetic pigment content was based on the method of Arnon [65] with slight modifications. 0.2 g of leaves were weighed and soaked in 95% ethanol for 24 h in dark environment, the absorbance values at 470 nm, 649 nm and 665 nm were determined spectrophotometrically and the content of each pigment was calculated using the formula.

### Determination of hormones content

The hormones content was determined using enzyme-linked immunosorbent assay (ELISA) kit (Jiangsu Su Enzyme Tech Biotechnology Co., Ltd.). The operation procedure was carried out with reference to the instruction manual of the kit.

### Determination of photosynthetic gas exchange parameters

Photosynthetic gas exchange parameters of flag leaves were determined between 8:00 and 11:00 using a portable photosynthesis system LI-6400 (Li-Cor, USA). The photosynthetically active radiation intensity was set at 1200 μmol m^–2^ s^–1^, the flow rate at 300 mL min^–1^, and the ambient CO_2_ concentration was controlled at 390–400 mmol L^–1^ using a buffer bottle. The net photosynthetic rate (P_n_), stomatal conductance (G_s_), transpiration rate (T_r_), and intercellular CO_2_ concentration (C_i_) were recorded after reaching a constant steady state with 10 biological replicates for each treatment.

### Sequencing and assembly

Sequencing libraries were created for the 36 samples and Illumina paired-end (PE) sequencing using the Illumina Novaseq 6000 platform was performed at Shanghai Majorbio Bio-pharm Technology, Shanghai, China, following the manufacturer’s instructions (Illumina, San Diego, CA). To ensure the production of high-quality clean reads, reads were were trimmed and quality controlled by fastp [66]. The mapped reads of each sample were assembled by StringTie in a reference-based manner [67].

### Differential expression analysis and Functional enrichment

RSEM [68] was used to quantify gene abundances. Differential expression analysis was performed using the DESeq2 [69]. DEGs with |log2FC|≧1 and FDR ≤ 0.05were considered to be significantly different expressed genes. The HISAT2 program [70] was used to align the clean reads to the rice reference genome (“Oryza_sativa”, http://rice.uga.edu/), providing information about the unique genomic loci and characteristics of the sequenced samples. In addition, functional-enrichment analysis including GO and KEGG were performed to identify which DEGs were significantly enriched in GO terms at *P*-value ≤ 0.05. GO functional enrichment and KEGG pathway analysis were performed utilizing Goatools and KOBAS [71].

### qRT-PCR

The HiScript II RT Reagent Kit (Vazyme, Nanjing, China) was used to reverse transcribe RNA samples to cDNA. Configure the degenome reaction system in a 200 μL RNase-free centrifuge tube. qRT-PCR was performed using gene-specific primers and SYBR Green Master Mix reagents (Vazyme, Nanjing, China) as well as the RoChe-LC480 system according to the manufacturer's instructions. The primers were designed with Primer Premier 5.0 and are listed in Table S7.

### Supplementary Information

Supplementary Material 1.

Supplementary Material 2.

Supplementary Material 3.

Supplementary Material 4.

Supplementary Material 5.

Supplementary Material 6.

Supplementary Material 7.

Supplementary Material 8.

Supplementary Material 9.

Supplementary Material 10.

Supplementary Material 11.

## Data Availability

All of the raw data used in this study have been deposited in NCBI (BioProject accession: PRJNA1020267, website: http://www.ncbi.nlm.nih.gov/bioproject/1020267). Experimental materials were stored in the Institute of Plant Resources and Environment, College of Life Sciences, Nanjing Normal University.
