# Supplementary material for: Comparative transcriptome analysis reveals major genes, transcription factors and biosynthetic pathways associated with leaf senescence in rice under different nitrogen application
Source: BMC Plant Biol. 2024 May 18;24:419. doi: 10.1186/s12870-024-05129-x (PMC11102181; doi:10.1186/s12870-024-05129-x)
Supplement: Supplementary file 8 — Supplementary Material 8. [file 12870_2024_5129_MOESM8_ESM.docx]

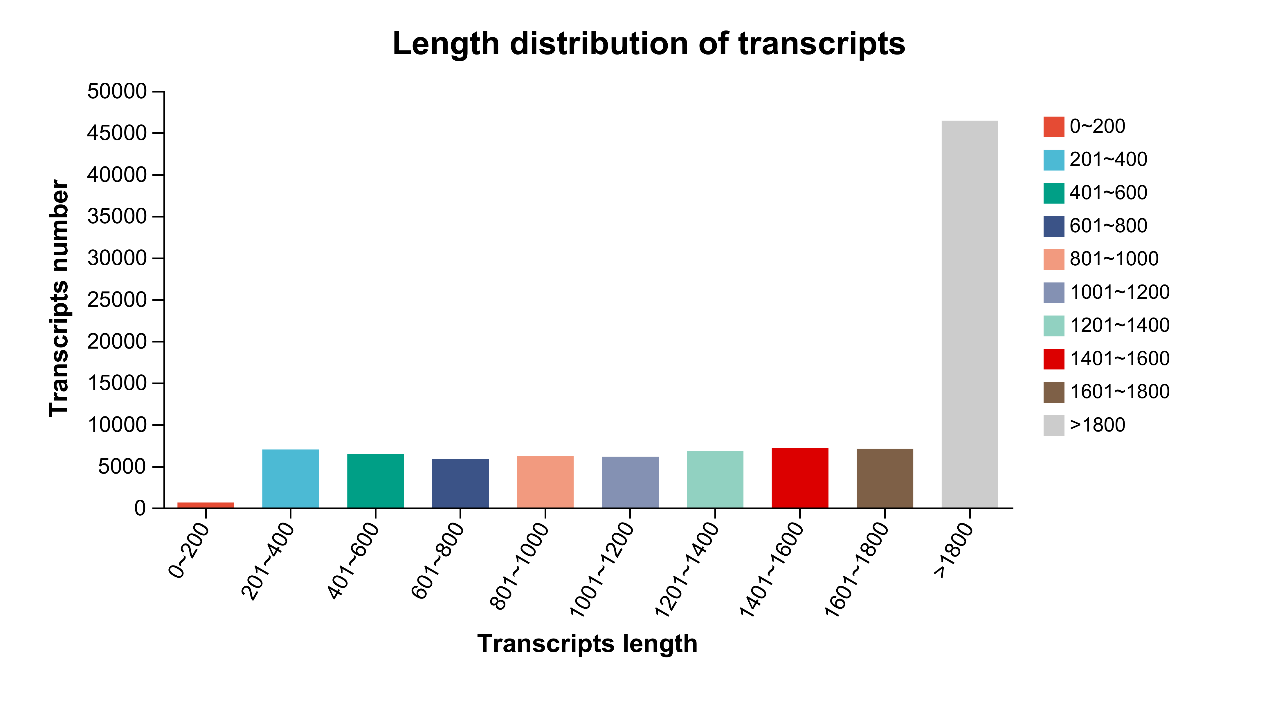


**Fig. S1** Distribution of different transcript lengths. The horizontal coordinate is the range of transcript lengths; the vertical coordinate is the number of transcripts within that range of transcript lengths.
