# Supplementary material for: Comparative transcriptome analysis reveals major genes, transcription factors and biosynthetic pathways associated with leaf senescence in rice under different nitrogen application
Source: BMC Plant Biol. 2024 May 18;24:419. doi: 10.1186/s12870-024-05129-x (PMC11102181; doi:10.1186/s12870-024-05129-x)
Supplement: Supplementary file 9 — Supplementary Material 9. [file 12870_2024_5129_MOESM9_ESM.docx]

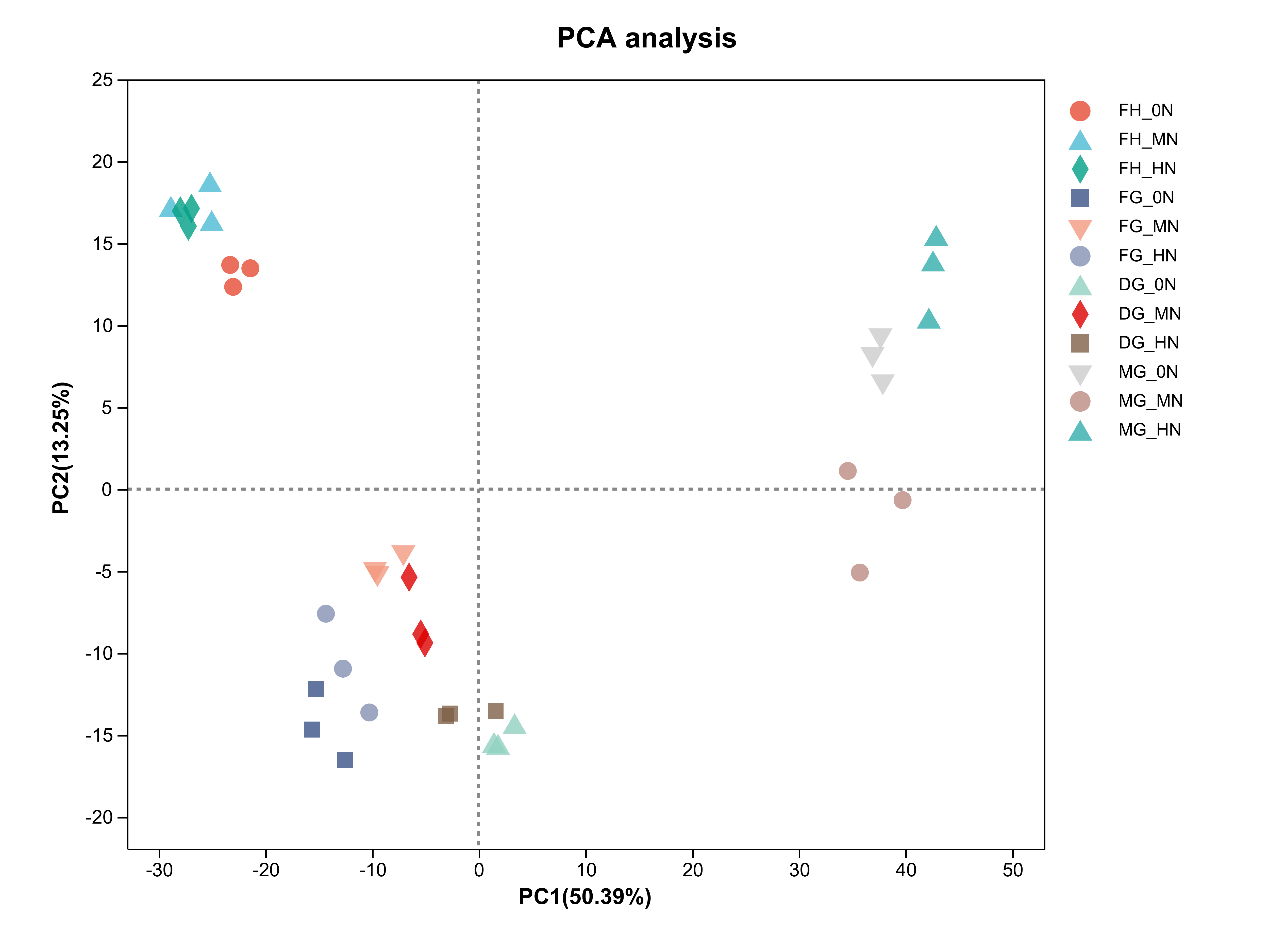


**Fig. S2** PCA of RNA-seq data of nitrogen application rates at four stages. The horizontal axis represents the contribution of Principal Component 1 (PC1) to discriminate the samples in the two-dimensional plot, and the vertical axis represents the contribution of Principal Component 2 (PC2) to discriminate the samples in the two-dimensional plot. The closer the distance of each sample point indicates the higher similarity between the samples.
