# Supplementary material for: Comparative transcriptome analysis reveals major genes, transcription factors and biosynthetic pathways associated with leaf senescence in rice under different nitrogen application
Source: BMC Plant Biol. 2024 May 18;24:419. doi: 10.1186/s12870-024-05129-x (PMC11102181; doi:10.1186/s12870-024-05129-x)
Supplement: Supplementary file 10 — Supplementary Material 10. [file 12870_2024_5129_MOESM10_ESM.docx]

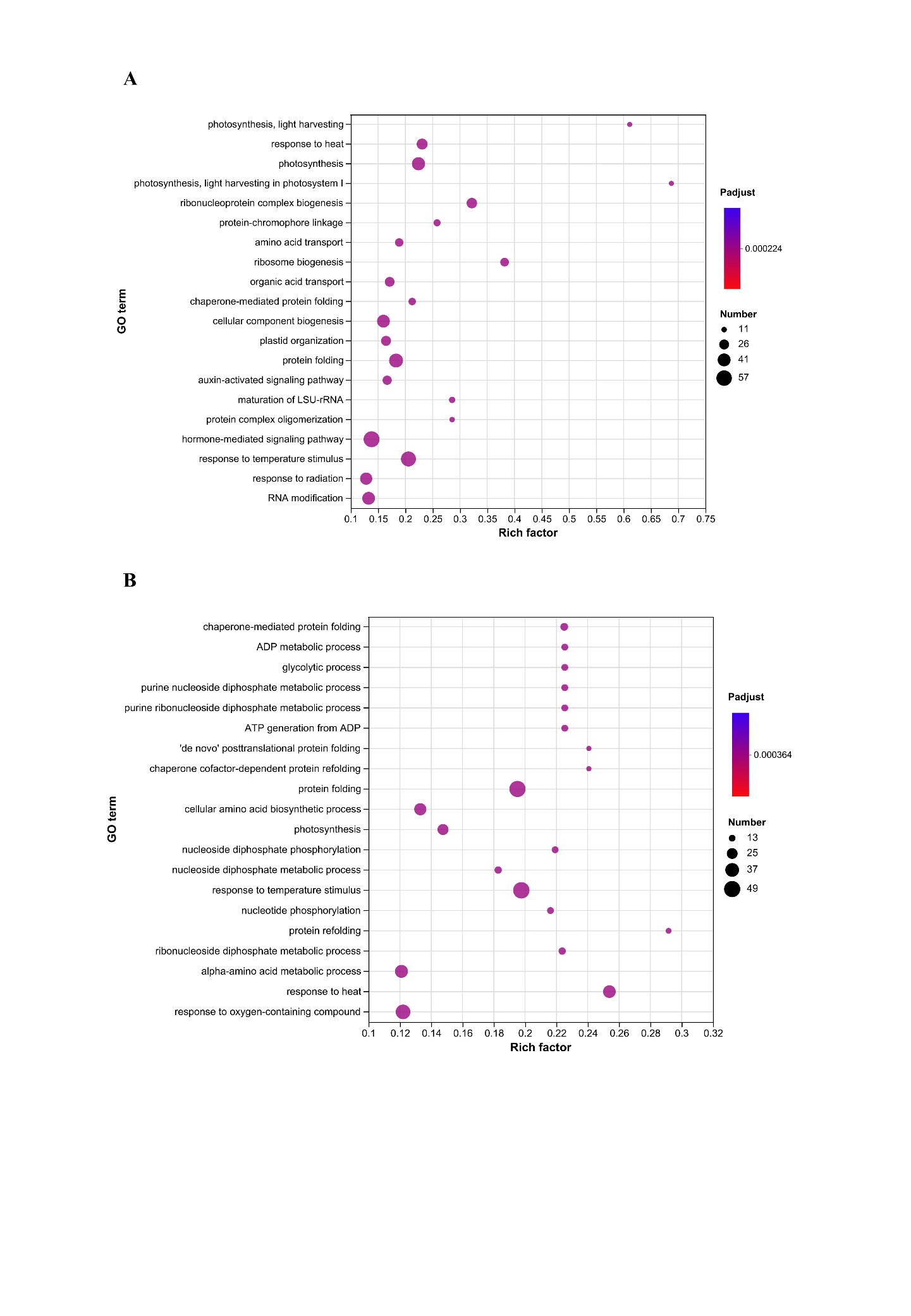


**Fig. S3** An overview of the GO terms signifcantly enriched in DEGs. A. GO enrichment of DEGs that up-regulated in 0N and down-regulated in HN conditions. B. GO enrichment of DEGs that up-regulated in HN and down-regulated in 0N conditions. The horizontal axis indicates the enrichment scores of DEGs, and the specifc pathways are plotted along the vertical axis. The color of each dot represents the corrected *P-value* for the corresponding pathway, and the dot size indicates the number of the DEGs associated with each corresponding pathway.
