# Supplementary material for: Comparative transcriptome analysis reveals major genes, transcription factors and biosynthetic pathways associated with leaf senescence in rice under different nitrogen application
Source: BMC Plant Biol. 2024 May 18;24:419. doi: 10.1186/s12870-024-05129-x (PMC11102181; doi:10.1186/s12870-024-05129-x)
Supplement: Supplementary file 11 — Supplementary Material 11. [file 12870_2024_5129_MOESM11_ESM.docx]

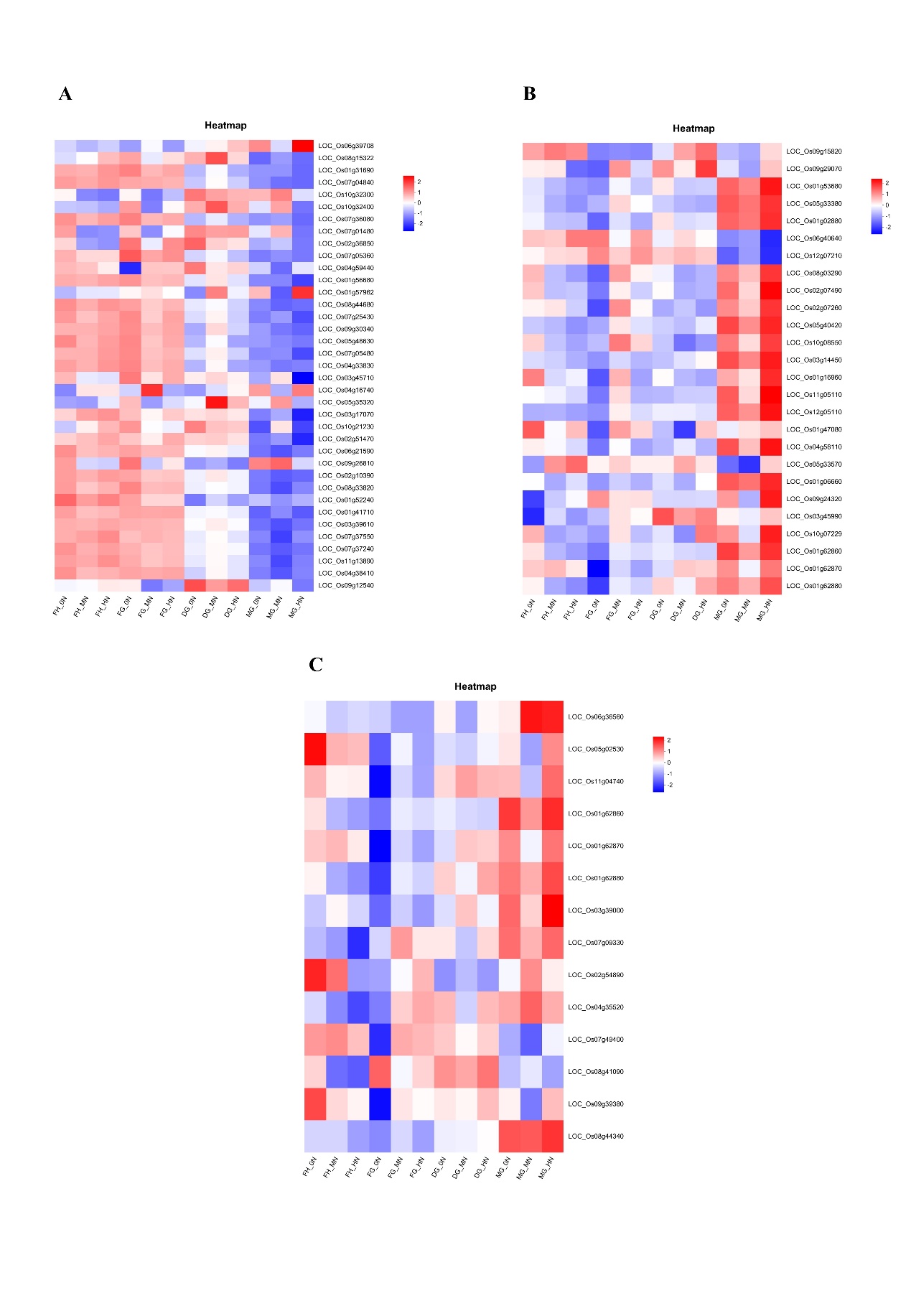


**Fig. S4** Key enzymes expressed in our study have colored shaded rectangles and genes link to these enzymes as well as their expression are presented in the heatmap. A. DEGs in photosynthesis pathway. B. DEGs in glycolysis/gluconeogenesis pathway. C. DEGs in ascorbate and aldarate metabolism pathway.
